# Supporting data sharing

**DOI:** 10.1038/s41523-019-0103-0

**Published:** 2019-02-13

**Authors:** Rebecca Kirk, Larry Norton

**Affiliations:** 1 Nature Partner Journals, Nature Research, 4 Crinan Street, London, N1 9XW UK; 2 Memorial Sloan-Kettering Cancer Center, New York, NY USA

To a degree that is remarkable if not startling, our means of communicating scientific information influences the actual process of investigation itself: what we study, how we conduct experiments and even our formulation of questions. To serve paper publishing, for example, with its physical constraints of space and two-dimensional presentation, we have needed to produce data that can be analyzed in such a way that the results can be communicated in conventional tables, figures and graphs as well as natural (linear) language. This forces a certain condensation or abstraction at best or an editing at worst, threatening to obscure subtleties of importance or even cause misinterpretation via errors of omission. As we have transitioned to digital formats we have most often just transferred the conventions that were appropriate for paper to our screens despite the vastly greater possibilities that the new medium offers.
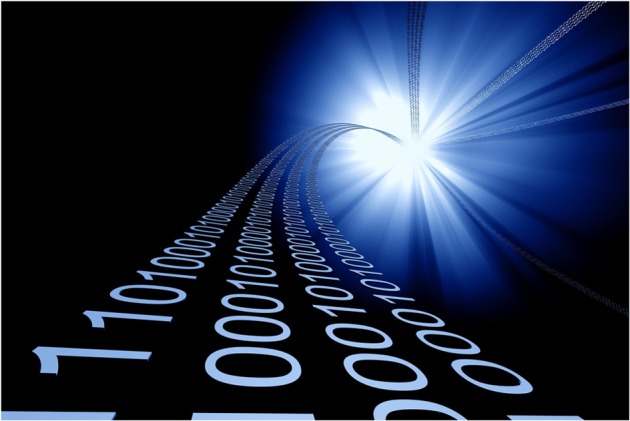


Yet, a most valuable product of any research study is the data themselves rather than just the summary of that data that results from processing it into ‘publishable’ form. The authors’ interrogation and interpretation of data is certainly valuable (for without that no conclusions could be reached). But, in too many cases that is the end of the story. The ‘closed’ data remains hidden behind the figures and graphs, out of reach of other minds and analytical tools. However, were we able to make actual data more accessible, this could open the door to further evaluation, deeper examination and, as time progresses, novel elucidations based on new discoveries and improved techniques of data handling and manipulation. For these reasons we believe that ‘open’ data should drive the science of the future. In addition, data sharing rewards data creators with amplified credit for their work.^1–4^

The evolution towards open data has already started in that it is now the norm that some types of data are routinely made publicly available when research is published. Examples include RNA sequencing information, DNA sequences and proteomics outputs.^5^ *npj Breast Cancer* is taking the next step. We will now be providing all our authors with additional support: editorial guidance to make their data, regardless of data type or degree of complexity, as open as is currently achievable. We will not be changing our data sharing policies, which remain in line with the Nature Research stable of journals and that require a data availability statement describing whether and how data may be accessed in all published manuscripts.^5^ However, we will be enabling authors of all accepted papers to ensure that their data, in as much detail as is feasible, be described fully and made accessible to the scientific community. To accomplish this, a Research Data Editor at Nature Research will work with authors to catalogue, label and annotate the datasets supporting their published work. This will create a metadata record for each published article and a detailed data availability statement to be included in their article.

The first example of this collaboration with our authors can be seen in the Data Availability statement in the recent publication from Sunil Kumar and colleagues^6^ and its associated metadata record^7^ in the journal-specific FigShare repository.^8^

The provision of this service for all of our authors is in part a response to the calls for broader data sharing that have been gathering pace in the cancer research community.^9^ It also builds on earlier work published in the journal that focused on signposting data resources to those working in the field.^10^ With our partner the Breast Cancer Research Foundation it is our intention to assist researchers in making progress against breast cancer via innovations in science publishing as well as scientific discovery. We hope and trust that our authors—and especially the readers of work published in our journal—will find the addition of clear data signposting for each manuscript to be of considerable value. And we encourage your feedback regarding our new initiative as well as your enthusiastic participation!
